# Carotenoid Supplementation Positively Affects the Expression of a Non-Visual Sexual Signal

**DOI:** 10.1371/journal.pone.0016326

**Published:** 2011-01-25

**Authors:** Alain J.-M. Van Hout, Marcel Eens, Rianne Pinxten

**Affiliations:** Research Group of Ethology, University of Antwerp, Wilrijk, Belgium; University of Lethbridge, Canada

## Abstract

Carotenoids are a class of pigments which are widely used by animals for the expression of yellow-to-red colour signals, such as bill or plumage colour. Since they also have been shown to promote immunocompetence and to function as antioxidants, many studies have investigated a potential allocation trade-off with respect to carotenoid-based signals within the context of sexual selection. Although an effect of carotenoids on non-visual (e.g. acoustic) signals involved in sexual selection has been hypothesized, this has to date not been investigated. First, we examined a potential effect of dietary carotenoid supplementation on overall song rate during the non-breeding season in captive male European starlings (*Sturnus vulgaris*). After only 3–7 days, we found a significant (body-mass independent) positive effect of carotenoid availability on overall song rate. Secondly, as a number of studies suggest that carotenoids could affect the modulation of sexual signals by plasma levels of the steroid hormone testosterone (T), we used the same birds to subsequently investigate whether carotenoid availability affects the increase in (nestbox-oriented) song rate induced by experimentally elevated plasma T levels. Our results suggest that carotenoids may enhance the positive effect of elevated plasma T levels on nestbox-oriented song rate. Moreover, while non-supplemented starlings responded to T-implantation with an increase in both overall song rate and nestbox-oriented song, carotenoid-supplemented starlings instead shifted song production towards (reproductively relevant) nestbox-oriented song, without increasing overall song rate. Given that song rate is an acoustic signal rather than a visual signal, our findings therefore indicate that the role of carotenoids in (sexual) signalling need not be dependent on their function as pigments.

## Introduction

Carotenoids are a class of pigments which are widely used by animals for the expression of yellow-to-red colour signals, such as bill or plumage colour, often within the context of sexual selection [1], [2]. It is frequently noted that carotenoid-based signalling may depend on the positive effects on immunocompetence and/or the anti-oxidative capacity that these pigments display ([3], [4]; but see [5]). As the latter two functions prevent carotenoids from being used as pigments (and vice versa), this dual function is thought to result in an allocation trade-off, which has been the focus of numerous studies [4], [6]. Interestingly, a small number of studies suggest that also non-carotenoid antioxidants can affect the expression of carotenoid-based sexual signals [7], [8], [9]. Moreover, two recent studies have reported a positive effect, respectively, of dietary carotenoid supplementation on paternal fanning behaviour in male three-spined sticklebacks (*Gasterosteus aculeatus*) [10] and of dietary supplementation of an antioxidant (specifically, vitamin E) on begging behaviour in yellow-legged gull chicks (*Larus michahellis*) [11]. It could, therefore, be hypothesized that the availability of carotenoids, for instance through their effect on immunocompetence or their antioxidative capacity, could also affect non-carotenoid-based signals within a sexual selection context. Although a potential positive effect of carotenoids on non-visual (e.g. acoustic) signals involved in sexual selection has already been proposed [12], to our knowledge, this has not yet been investigated.

Testosterone (T) is a major hormone involved in the expression of many sexual signals, including both carotenoid-based signals as well as signals that are not dependent on carotenoid pigmentation, such as song rate [13], [14], [15]. It has been proposed that elevated plasma T reduces immunocompetence and elevates oxidative stress, the latter possibly through its effect on metabolic rates [4], [16], [17]. As carotenoids display an antioxidative capacity, they could potentially alleviate these negative effects [4]. Additionally, some evidence suggests that T can increase carotenoid absorption capacity in the intestine [18] and can positively affect plasma levels of lipoproteins, which can function as carriers of carotenoids [19]. Due to the above, an interaction between carotenoids and T could therefore be expected [3], [4], [6]. However, to our knowledge, it is not known whether a possible interaction between plasma T levels and carotenoid availability (for instance via oxidative stress) could also affect non-carotenoid-based signals involved in sexual selection.

The present study was performed on male European starlings (*Sturnus vulgaris*), a seasonally breeding songbird in which males produce high levels of song throughout most of the year, even during fall, when plasma T levels are basal [14]. While it has been shown that during the breeding season starling song is involved in female mate choice and in male-male interactions [20], [21], [22], [23] and that song rates may affect fitness (reviewed by [23]), it has been suggested that non-breeding song may also affect mating decisions and pair formation, as well as social cohesion [24]. As previous studies suggest that carotenoids may affect signals that are not dependent on carotenoid colouration [10], [11], we hypothesized that elevated plasma carotenoid levels would positively affect song rates in European starlings during fall (non-breeding season) and tested this hypothesis using a dietary carotenoid supplementation experiment. Furthermore, it has also been demonstrated in starlings as well as other song bird species, that experimentally elevating plasma T levels during fall (when T levels are basal) induces reproductive behaviours, typical for the breeding season, such as nestbox/territory occupation and nestbox-oriented song expression [13], [25], [26]. Therefore, in the second part of this study, we investigated whether carotenoid supplementation affects the modulation by plasma T levels of nest-oriented song expression, as well as nestbox occupation behaviour and overall song production. To this end, using the same birds, we subsequently performed a T-implantation experiment after the start of the carotenoid supplementation. By performing our experiment during fall, when plasma T levels are basal, we were able to first examine a potential effect of carotenoid supplementation on song rate while excluding a possible interaction with the elevated plasma T levels typical of the breeding season. Subsequently, this allowed us to separately and experimentally examine the potential effect of carotenoid supplementation on T-induced song behaviour. In combination, we were therefore able to examine in greater detail how carotenoid availability and plasma testosterone levels may interact in their effects on song behaviour in European starlings.

## Materials and Methods

### Ethics statement

The housing and experimental procedures were performed in agreement with the Belgian and Flemish laws and were approved by the ethical committee of the University of Antwerp (ID number 2006/22). European starlings have been shown to adapt easily to captivity and to show normal social behaviour under these conditions [21], [27]. Both aviaries used in this study were equipped with 20 identical nestboxes, each equipped with a 30 cm perch allowing the males to sit or sing in front of it, several larger perches, shelter, and food and water ad libitum. Implantation of Silastic™ tubes (Degania Silicone) was performed under localized anaesthesia. When performing the implantations and when taking blood sampling, care was taken to minimize stress for the starlings.

### Origin and Housing of Starlings

The 37 male starlings used in this study had been captured as juveniles and were then housed on the grounds of Campus Drie Eiken of the University of Antwerp (Wilrijk, Belgium) in large single sex outdoor aviaries (but note that free-living starlings frequently visit these grounds). At the time the study took place they were between 2 and 4 years old and were (pseudorandomly distributed) housed in two aviaries (L × W × H: 16×8×2.4 m (N = 18 starlings) and 17.5×7×2.75 m (N = 19 starlings)).

### Carotenoid supplementation and testosterone implantations

Starting on 26 November 2008, half of the starlings (N = 19, i.e. one aviary) received dietary carotenoid supplementation, while the other half did not (N = 18). The dietary carotenoid supplementation consisted of addition of 50 g of ORO GLO® brand 15 Dry Pigmenter (Kemin, USA; extracted from marigolds (*Tagetes erecta*) and containing 15.0 grams of xanthophyll per kg, of which 85% lutein) per 1 kg of the starlings' standard feed (mixed 1/3 Orlux Uni patee, Orlux, Belgium and 2/3 Merelkorrel Speciaal, Nifra - Van Camp, Belgium). Taking into account the average daily consumed quantity of feed and the number of starlings in the aviary, this corresponds to an average daily dose per individual of approximately 10 mg, which can be considered moderate [28]. Although information on plasma carotenoids is, to our knowledge, not available for European starlings, a recent study on the closely-related spotless starling (*Sturnus unicolor*) showed that lutein represented the largest fraction of plasma carotenoids in this species [29]. On 2 and 3 December 2008, half of the carotenoid-supplemented and half of the non-supplemented starlings received a T-filled implant in their left flank (N = 10 and N = 9, respectively; 15 mm Silastic™ tubes, Degania Silicone: i.d.: 1.47 mm; o.d.: 1.96 mm; see e.g. [14]), while the remaining birds received empty implants (for both N = 9). Numerous previous studies have proved the efficacy of this method for increasing T levels from baseline up to breeding levels in European starlings, both in the wild and in captivity [13], [14], [25], [26]. Blood samples (of approximately 500 µL) were collected and body mass was measured one day before the start of the carotenoid supplementation, as well as one week thereafter (i.e. immediately before the T-implantations) and a third time one week after the T-implantations.

### Lutein assays

The concentration of lutein was measured following [30]. Twenty microliter of plasma was diluted in 180 µl of absolute ethanol. The sample was then mixed on a vortex for 1 minute and subsequently centrifuged at 4°C at 1,500 g for 10 minutes. The absorbance of the supernatant was measured at 445 nm. The overall lutein concentration in the plasma was calculated using the average extinction coefficient for lutein of E_1cm_^1%^ = 2550 in ethanol and a molecular mass of 569 g/mol. It has been demonstrated that this colorimetric technique is highly correlated with HPLC measurements of carotenoid concentration of the plasma and can therefore be considered as a representative technique [30].

### Observations

Using a point sampling technique, we monitored behaviour of all starlings within one aviary simultaneously, in (uninterrupted) sessions of approximately 45 min, with an interval of one minute (e.g. [14]), all between 09h00 and 13h00. Behavioural observations were made during 3 different periods: 1) 1–4 days prior to the carotenoid supplementations, 2) 3–7 days after the start of the carotenoid supplementation, and 3) 11–15 days following the T-implantations. During each period, observations were performed on 3 days. Per day, one observation session for each aviary was performed, while alternating the order of the aviaries between subsequent days. Overall song rate was defined as the proportion of samples during which a male was singing (in any and all locations) compared to the total number of samples. Furthermore, we also determined the proportion of the time spent inside a nestbox (which may or may not involve singing) as well as the nestbox-oriented song rate, which only includes song production inside the nestbox or on the perch in front of the nestbox [13]. It has previously been shown in European starlings that (overall) song rate is repeatable, even between seasons (r = 0.5) [31]. This is confirmed using data from this study: overall song rate, r = 0.22, p<0.0001; proportion of time spent inside a nestbox after T implantation, r = 0.12, p = 0.01; nestbox-oriented song rate, r = 0.32, p<0.0001 [32].

### Statistical analyses

Statistical analyses were performed in SAS™ (version 8). Data were tested for normality using Shapiro-Wilk tests, and log-transformed when necessary (i.e. for plasma lutein levels and the time spent inside a nestbox). A three-way repeated measures mixed model with interaction effects was used to test the effect of carotenoid supplementation, T-implantation and period (as defined above) on plasma lutein levels. Another three-way mixed model was used to examine the effects of carotenoid supplementation and T-implantation (as well as their interaction) on the proportion of body mass during the second and third period (see above) compared to before the start of carotenoid supplementation.

The proportional behavioural data were first arcsine-square root transformed. For the first part of the study, which investigated effects on overall song rate, a two-way design with carotenoids supplementation, and period (i.e. before versus after the start of supplementation), together with their interaction, was used. For the second part of the study, a three-way design with carotenoids supplementation (Caro-supplementation from heron), T-implantations and period, together with their interactions, was used to investigate overall song rate, nestbox-oriented song rate and the proportion of time spent inside a nestbox. This resulted in comparisons between 4 groups: sham-implanted non-supplemented starlings (C-group), T-implanted non-supplemented starlings (T-group), sham-implanted Caro-supplemented starlings (CaroC-group), T-implanted Caro-supplemented starlings (CaroT-group). For all post hoc comparisons, the Tukey and Tukey-Kramer corrections were employed (probabilities mentioned as p_a_). Unless otherwise specified, values reported are means ± SE. For all tests, an α of 0.05 was used to judge significance.

## Results

### Plasma lutein levels and body mass

**Figure 1:**
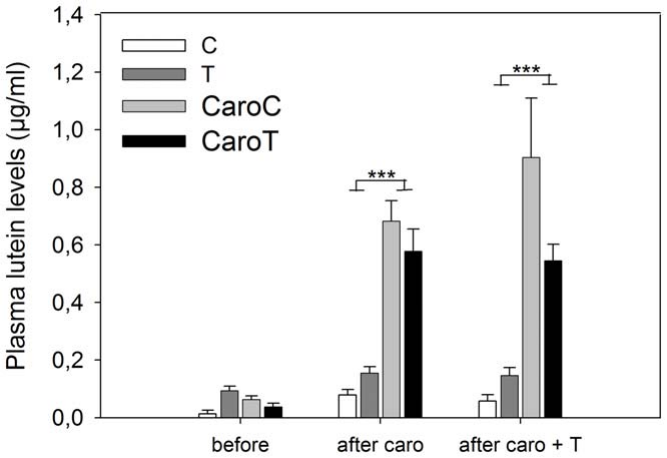
Plasma lutein levels (means ± SE) before carotenoid supplementation, after carotenoid supplementation and after carotenoid supplementation + T-implantation. Non-supplemented groups: sham-implanted (C – white) and T-implanted (T – dark grey), and the carotenoid-supplemented groups: sham-implanted (CaroC – light grey) and T-implanted (CaroT – black) (CaroT: N = 10, all others: N = 9). Significant differences within periods are shown: ***p<0.001.

No general effect of T-implantation on plasma lutein levels was observed, nor an effect of T within either the supplemented or the non-supplemented starlings separately (CaroC versus CaroT and C versus T, respectively; mixed model, all p_a_≥0.9). These factors were therefore removed from the model. There was a significant interaction effect between carotenoid supplementation and period (mixed model, F_2,67_ = 22.21, p<0.001). Post hoc tests showed that while no significant difference was observed before Caro-supplementation (t_67_ = 1.23, p_a_ = 0.8), plasma lutein levels were significantly higher in Caro-supplemented than in non-supplemented starlings one week after the start of Caro-supplementation (mixed model, t_62_ = −6.64, p_a_<0.0001; Figure 1), as well as one week after the T-implantations (t_62_ = −7.35, p_a_<0.0001).

A three-way mixed model showed no significant effect of the T-implantations on body mass (t_33_ = −0.54, p_a_ = 0.9), nor a significant three-way interaction effect between T-treatment, Caro-treatment and period (F_1,33_ = 0.38, p = 0.5). These factors were therefore removed from the model. Hereafter, we found a significant positive effect of Caro-supplementation on body mass (mixed model, F_1,35_ = 18.06, p<0.001), and a nearly significant interaction effect between period and Caro-treatment (F_1,33_ = 3.83, p = 0.058). Post hoc tests showed that body mass was significantly higher in Caro-supplemented than in non-supplemented starlings one week after the T-implantations (t_35_ = −4.39, p_a_<0.001; 99.4±1.0% and 95.1±1.0%, respectively), while this was not (yet) the case one week after the start of Caro-supplementation (t_35_ = −1.62, p_a_ = 0.4; 100.8±1.0% and 98.5±1.0%, respectively).

### Effects of carotenoid supplementation on non-breeding song rate

**Figure 2:**
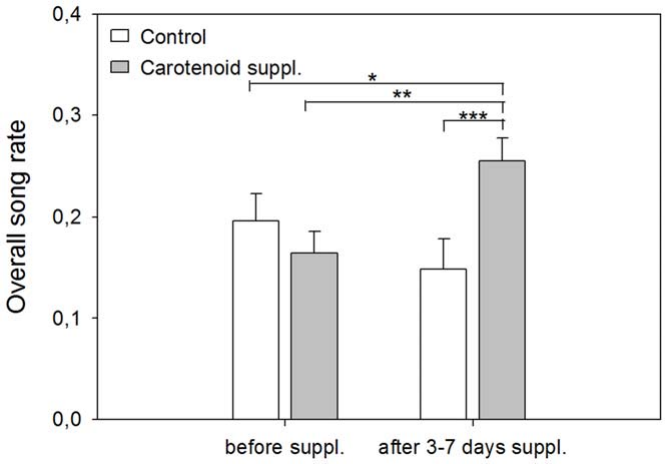
Overall song rate (means ± SE) before and after the start of carotenoid supplementation. Non-supplemented starlings (white; N = 18) and carotenoid-supplemented starlings (grey; N = 19). *p<0.05, **p<0.01, ***p<0.001.

A model examining overall (non-breeding) song rate, resulted in a significant interaction effect between period (i.e. before versus after the start of Caro-supplementation) and group (F_1,35_ = 16.77, p<0.001). Post hoc tests showed that after Caro-supplementation (t_35_ = −4.44, p<0.01; Figure 2), but not before Caro-supplementation (t_35_ = 1.35, p = 0.5), song rate of the Caro-supplemented group was significantly higher than that of the non-supplemented group.

### Interaction between carotenoids and testosterone

**Figure 3:**
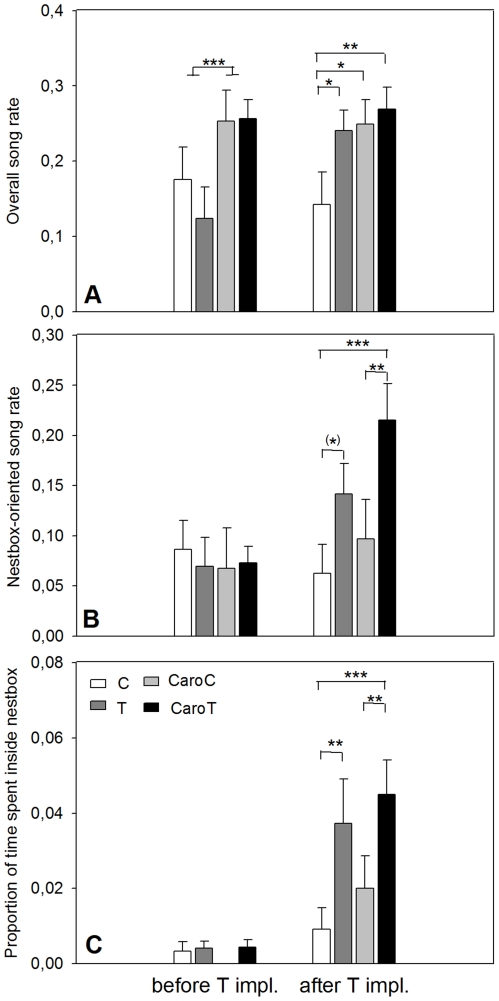
Overall song rate, nestbox-oriented song rate and time spent inside nestbox (means ± SE), before and after T-implantation. Non-supplemented groups: sham-implanted (C – white) and T-implanted (T – dark grey), and the carotenoid-supplemented groups: sham-implanted (CaroC – light grey) and T-implanted (CaroT – black) (CaroT: N = 10, all others: N = 9). Significant differences within periods are shown: (*)0.05<p<0.10, *p<0.05, **p<0.01, ***p<0.001.

For overall song rate, there was a significant three-way interaction effect between T-treatment, Caro-treatment and period (F_2,33_ = 3.64, p = 0.03). Post hoc tests show that prior to the T-implantations overall song rate was significantly higher in Caro-supplemented than in non-supplemented starlings (t_33_ = −5.12, p_a_<0.0001). After the T-implantations, the C-group displayed a significantly lower song rate (all p_a_<0.05; Figure 3A) than the 3 other groups, which had comparable song rates (all p_a_ = 1.0).

For nestbox-oriented song rate, no significant three-way interaction effect between T-treatment, Caro-treatment and period was found (F_1,33_ = 1.0, p = 0.4). Nestbox-oriented song rate showed a significant interaction effect between T-treatment and period (mixed model, F_1,33_ = 11.81, p<0.01; Figure 3B), with nestbox-oriented song rate being significantly higher in T-implanted than in sham implanted starlings after (t_33_ = −5.01, p_a_<0.0001) but not before the T-implantations (t_33_ = −0.15, p_a_ = 1.0). Similarly, there was a nearly significant interaction effect between Caro-supplementation and period (mixed model, F_1,33_ = 2.96, p = 0.09), with nestbox-oriented song rate being nearly significantly higher in Caro-supplemented (CaroC and CaroT) than in non-supplemented (C and T) starlings after the T-implantations (t_33_ = −2.58, p_a_ = 0.07) but not before the T-implantations (t_33_ = −0.15, p_a_ = 1.0).

To facilitate graphical representation, three-way post hoc comparisons were used in Figure 3: after the T-implantations nestbox-oriented song rate did not significantly differ between the T and the CaroT starlings (t_33_ = −2.32, p = 0.3) nor between the C and CaroC starlings (t_33_ = −1.36, p_a_ = 0.9) or T and CaroC (t_33_ = −1.72, p_a_ = 0.7). Nestbox-oriented song rate of CaroT starlings was however significantly higher than that of C starlings (t_33_ = −5.37, p_a_<0.001) and that of CaroC starlings (t_33_ = −3.98, p_a_<0.01). Nestbox-oriented song rate also displayed a tendency to be higher in the T than in the C starlings (t_33_ = −3.11, p_a_ = 0.07).

For the proportion of time spent inside a nestbox a similar pattern as that of nestbox-oriented song rate is found, except that the difference after the T-implantations between Caro-supplemented and in non-supplemented starlings did not reach the significance level to be considered a trend (t_33_ = −2.35, p_a_ = 0.11; Figure 3C).

## Discussion

The relationship between carotenoids, carotenoid-based sexual signals and/or T has received much attention from researchers, particularly in recent years [1], [2], [4], [15]. Notably, a potential effect of antioxidants on acoustic signals involved in sexual selection has recently been suggested [12], although the authors focussed on potential effect of carotenoids on developmental processes rather than on direct effects on song production during adulthood. To our knowledge, our study is the first to investigate (and observe) a possible effect of carotenoid availability on a non-visual signal involved in sexual selection, i.e. song rate in European starlings, and to investigate whether carotenoids affect the modulation of such a signal by plasma T levels.

### Carotenoid supplementation and overall song rate

Our results indicate that, during the non-breeding season, song rate in adult male European starlings was significantly elevated less than one week after starting Caro-supplementation, compared to non-supplemented starlings. Although we are not aware of information on plasma lutein levels in wild European starlings, plasma lutein levels observed in our Caro-supplemented group were well below those recently measured in the closely-related spotless starling (*Sturnus unicolor*; ca. 5.6 µg/ml; [29]). It is therefore unlikely that the plasma levels measured in our study subjects are abnormally high. Furthermore, the timeframe of this effect of Caro-supplementation on song rate appears to be comparable to that of antioxidants on begging rate in gull chicks (2 days; [11]) and of carotenoids on flight performance in American goldfinches (*Carduelis tristis*) (7 days; [33]).

As a potential mechanism for the observed positive effect of Caro-supplementation on overall song rate, increased plasma carotenoid levels may have generally alleviated the negative effects of increased oxidative stress due to the metabolic demands of higher song rates [34], [35]. Conversely or additionally, Caro-supplementation may have specifically alleviated elevated oxidative stress in the vocal musculature, similarly to what has been observed for flight musculature [36]. Furthermore, studies have also demonstrated a positive effect of carotenoids on immunocompetence [1], [18], [30]. In combination with an observed positive correlation in European starlings between song rate and (cell-mediated) immunity [37], this offers an alternative or complementary hypothesis to the one above. Further research is however needed to test these hypotheses. Additionally, it could be hypothesized that carotenoid supplementation may have elevated plasma T levels, which in turn positively affected song rate. However, this is contradicted by a lack of effect on nestbox-oriented song rate or on proportion of time spent inside a nestbox (which are both affected by T: see [13], [25], [26] and the second part of this study), as well as by the lack of a significant interaction effect between T-treatment and Caro-treatment on plasma T levels (C: 1.4±0.4, T: 4.4±0.4, CaroC: 1.7±0.5, CaroT: 5.0±0.8; Caro-supplementation: t = 0.828, p = 0.4 and T-implantation t = −4.047, p<0.001 and interaction effect: t = −0.343, p = 0.7).

When compared to the non-supplemented starlings, we also observed a positive effect of Caro-supplementation on body mass in the carotenoid-supplemented starlings (but do note that this was only significant at the end of the study). This is contrary to other studies which showed no effect or a negative effect of Caro-supplementation on body mass [2], [28], [30]. However, one study did show a positive effect on body mass of Caro-supplementation, when combined with supplementation with melatonin, a non-pigmentary antioxidant [7]. Although the positive effect on song we observed may also have resulted from increased feeding through a preference for carotenoid-rich food [38], this was not measured. Importantly, although carotenoid supplementation positively affected both overall song rate and body mass, the increase in overall song rate (after 3–7 days) preceded any significant body mass changes (i.e. these were significant after 2 weeks, but not yet after 1 week). It is therefore more likely that, although increased body mass may enhance song rate [39], the observed positive effect of Caro-supplementation on song rate in our study is not contingent on body mass changes. This effect therefore appears to be robust.

Although our results could potentially be attributed to an aviary effect, we think that this is unlikely: the rapid positive effect of Caro-supplementation on overall song rate was consistent with previously observed effects of carotenoids on (behavioural) performance (begging rate: [11]; flight performance [33]). Furthermore, data from the second part of our study show that, while overall song rate significantly differed between Caro-supplemented and non-supplemented starlings before T-implantation, nest-box oriented song rate or time spent inside a nestbox did not. Moreover, while the latter two traits were subsequently elevated after T implantation (as expected: [13], [25], [26]), overall song rates in both the non-supplemented and Caro-supplemented control groups (i.e. C and CaroC) remained stable.

### Interaction between testosterone treatment and carotenoid availability

Past studies in European starlings (as well as other species) have demonstrated that experimentally elevating plasma T levels up to breeding levels induces reproductive behaviour, such as increased song rate and increased nestbox visitation [13], [25], [26]. Our results show that two weeks after the T-implantations (and 3 weeks after the start of Caro-supplementation) nestbox-oriented song rate as well as nestbox visitation (i.e. time spent inside a nestbox) were significantly positively affected by T-implantation (i.e. T versus C and CaroT versus CaroC). Therefore, our results confirm that in this species mate attraction behaviour (in relation to nestbox occupation) is modulated by plasma T levels. Although our results did not show a significant effect of carotenoid availability on this pattern, the positive effect of T on nestbox-oriented song rate was highly significant in the Caro-supplemented groups, while the non-supplemented group only showed a tendency of the same effect. Therefore, although further research is required, a possible interpretation of our results could be that carotenoid availability enhances the positive effect of elevated plasma T on nestbox-oriented song rate in European starlings. If confirmed by future research, this may suggest that males which are able to maintain higher plasma carotenoid levels (through e.g. greater foraging efficiency or specificity [38] or physiological uptake efficiency [18]), would be able to display higher rates of reproductive song during the breeding season, to attract females and/or repel competing males [20], [21], [22], [23].

Interestingly, while in non-supplemented starlings T-implantation elicited an increase in both nestbox-oriented song rate and overall song rate, in Caro-supplemented starlings an increase in nestbox-oriented but not in overall song rate was observed. This suggests that while non-supplemented starlings responded to T-implantation with an overall increase in song production (including nestbox-oriented song), Caro-supplemented starlings instead shifted song production towards (reproductively relevant) nestbox-oriented song. This may be explained by an apparent upper limit in song rate, since the highest mean *group* song rates observed in this study are in the upper range of what has been observed in other studies using male starlings, both in the wild and in captivity [13], [25], [26], [27].

### Conclusions

By performing our experimental study during fall, we were able to show a positive effect of carotenoids on overall song rate that is not dependent on the high plasma T levels typical for the breeding season. Moreover, our results suggest that carotenoid supplementation enhanced the positive effect of subsequent T treatment on nestbox-oriented song rate. This indicates that elevated carotenoid availability could positively affect song rate both inside and outside the breeding season. Taken together, our results indicate that the interaction between song rate, carotenoids and T in European starlings is complex and deserves further study. Importantly, given that song rate is an acoustic signal rather than a (carotenoid-dependent) visual signal, our findings indicate that the role of carotenoids in (sexual) signalling need not be dependent on their function as pigments. Therefore, the interaction between carotenoids and signals involved in sexual selection may be more general than previously assumed.
